# Treatment of subclinical hyperthyroidism: effect on left ventricular mass and function of the heart using magnetic resonance imaging technique

**DOI:** 10.1530/EC-14-0137

**Published:** 2015-01-24

**Authors:** Peter D Mark, Mikkel Andreassen, Claus L Petersen, Andreas Kjaer, Jens Faber

**Affiliations:** 1 Department of Medicine O, Centre of Endocrinology and Metabolism, Herlev University Hospital, Herlev Ringvej 75, Herlev, DK-2730, Denmark; 2 Department of Clinical Physiology and Nuclear Medicine, Frederiksberg Hospital, Nordre Fasanvej 57, Frederiksberg, 2000, Denmark; 3 Department of Clinical Physiology Nuclear Medicine and PET, Rigshospitalet, Blegdamsvej 9, København Ø, Denmark; 4 Faculty of Health Sciences, University of Copenhagen, Blegdamsvej 3B, København N, 2200, Denmark; 5 Center for Functional and Diagnostic Imaging and Research, Hvidovre University Hospital, Kettegård Allé 30, Hvidovre, 2650, Denmark

**Keywords:** subclinical hyperthyroidism, treatment, cardiac effects, MRI

## Abstract

**Purpose:**

The aim of this study was to investigate structure and function of the heart in subclinical hyperthyroidism (SH) before and after obtaining euthyroidism by radioactive iodine treatment, using high precision and observer-independent magnetic resonance imaging (MRI) technology.

**Methods:**

Cardiac MRI was performed before and after euthyroidism was obtained by radioactive iodine treatment in 12 otherwise healthy patients (11 women and one man, mean age 59 years, range 44–71 years) with a nodular goiter and SH, and compared with eight healthy controls investigated at baseline. Cardiac data were expressed as an index, as per body surface area, except for heart rate (HR) and ejection fraction.

**Results:**

Post-treatment cardiac MRI was performed in median 139 days after a normalized serum TSH value had been recorded. During treatment, serum TSH increased from (median (range)) 0.01 (0.01–0.09) to 0.88 (0.27–3.99) mU/l. Patients with untreated SH had increased resting HR (*P*<0.01) as well as cardiac index (cardiac output as per body surface area) (*P*<0.01) compared with controls. Obtaining euthyroidism resulted in a significant decrease in left ventricular mass index (LVMI) of 2.7 g/m^2^ (*P*=0.034), in HR of 8 bpm (*P*=0.001), and in cardiac index of 0.24 l/min per m^2^ (*P*=0.017).

**Conclusions:**

Normalization of thyroid function by radioactive iodine treatment of SH resulted in significant reductions in clinically important heart parameters such as LVMI, HR, and cardiac index. SH should be regarded as a condition in which aggressive treatment should be considered to protect cardiac function.

## Background

Subclinical hyperthyroidism (SH) is defined by the biochemical pattern of reduced or undetectable serum thyroid-stimulating hormone (TSH) levels and thyroid hormone levels within the reference range. SH is seen with increasing frequency in the elderly and can be divided into either endogenous causes most often due to autonomous nodular thyroid disease, or exogenous causes due to suppressive therapy with thyroid hormones in thyroid cancer or goiter or overtreatment of hypothyroidism.

The symptoms in SH are vague, but adverse effects seem most prominent concerning the heart and the bones. Thus, SH increases bone loss by ∼50–100% in postmenopausal women due to increased bone turnover (1).

Concerning the heart, SH predisposes to the development of atrial fibrillation. In a recent large cohort from the Danish population, this effect seems to be present even among euthyroid people with serum TSH levels in the lower normal range, suggesting a dose-dependent manner of effect of slightly increased thyroid function on the heart (2).

The effects of SH on heart structure and function have been studied mainly by echocardiography, and, overall, the results show subtle changes, primarily increased left ventricular mass index (LVMI, LVM as per body surface area), and increased stiffness of the heart although data derived from studies on exogenous SH should be taken with care due to the fact that many of these patients were biochemically overt hyperthyroid. Echocardiography is observer dependent during the process of performing the investigation, and the procedure is less precise having a relatively high intra- and inter-observer coefficient of variation (CV) values. This results in the demands of testing large sample sizes. In contrast, magnetic resonance imaging (MRI) technology has a high precision and is observer independent during the imaging procedure (3). Holter monitor studies have in general demonstrated increased heart rate (HR), and three studies demonstrate increased HR in endogenous SH (4, 5, 6).

We have previously evaluated cardiac performance before and after radioactive iodine treatment of SH by means of the impedance cardiography technique, aiming at normalization of serum TSH levels, and found a reduction in both HR and cardiac output (CO) (7).

Subtle increases in HR and CO might imply a long-term consequence of increased cardiovascular disease and mortality (8, 9). Several meta-analyses have found increased mortality in SH (10, 11, 12, 13) and we have recently in a very large cohort of people from the general population demonstrated a 22% increased mortality and 9% increase in major cardiovascular events (MACEs) among subjects with SH (14).

Thus, we set out to test the effect of obtaining euthyroidism by means of radioactive iodine treatment given to otherwise healthy subjects with a nodular goiter and SH on heart morphology and function, as measured by cardiac MRI. During our study, an echocardiography study was published on 44 patients with SH who were treated with radioactive iodine (15). This study demonstrated significant reductions in CO and LVMI without changes in left ventricular ejection fraction (LVEF), and served to underscore the value of the present MRI-based study.

## Methods

Consecutive and otherwise healthy patients with endogenous SH due to a nodular goiter as evaluated by a thyroid scintigraphy were included. Inclusion criteria were as follows: serum TSH <0.10 mU/l repeated within 3 months, combined with serum thyroxine (T_4_) and tri-iodothyronine (T_3_) levels within the reference range. Exclusion criteria were as follows: positive TSH receptor antibodies, known cardiovascular disease including hypertension, any medication potentially affecting heart function, resting blood pressure (BP) >140/90 mmHg, and any other chronic diseases. The indication for radioactive iodine treatment was based on a combination of the finding of SH and compression symptoms from their goiter. Thus, the decision of treatment was based on accepted clinical practice at our institution and made it impossible to include a control group prospectively.

Fifteen patients were studied; however, three were excluded (one developed Graves' disease after radioactive iodine, one could not tolerate the MRI apparatus, and one developed early hypothyroidism after one treatment with radioactive iodine). This left us with 12 patients, 11 women and one man, aged 44–71 years (mean, 59.3±8.9).

We included a control group with regard to baseline data: eight healthy, unmedicated subjects (seven women and one man) aged 42–65 (mean, 55.4±7.5) were selected as matched controls, among employees at our institution. These subjects were matched on the basis of sex, age, and body surface area.

Radioactive iodine was given as one of the two doses: ∼400 or 600 MBq, the choice depending on the size of goiter as evaluated manually. All subjects should have a 24-h radioactive iodine uptake of >20%. After radioactive iodine treatment, thyroid function was followed by blood sampling after 1, 2, 5, and 8 months on a routine basis, and further testing was performed when necessary. Blood sampling were performed in the non-fasting state during 0800–1000 h. Cardiac MRI was performed at baseline on matched controls. Cardiac MRI was performed on subjects with SH before and after radioactive iodine treatment when serum TSH levels were normalized (defined as ≥0.20 mU/l), and at least 4 months after radioactive iodine treatment. The following time intervals were recorded: i) time from radioactive iodine treatment to normalization of serum TSH levels and ii) time from normalization of serum TSH levels to post-treatment cardiac MRI.

Cardiac MRI was performed on a 1.5 T whole-body scanner (Gyroscan ACS-NT, Philips Medical Systems, Best, The Netherlands) using a phased-array cardiac coil (Synergy, Philips Healthcare, Eindhoven, The Netherlands). Following localization of the long axis of the heart, contiguous true short-axis slices were acquired using breath-hold, electrocardiogram (ECG)-triggered cine-MRI. Each slice was obtained during one breath-hold of ∼20 s. Typically, the heart was covered by 10–15 slices of 10 mm. The number of phases obtained was 15–20 depending on HR. The temporal resolution was 46 ms. The field of view was 300 mm with a matrix of 256×256. Turbofield echo was used and sequence parameters were repetition time (TR), 9.6 ms, echo time (TE), 4.5 ms, and flip angle (FA), 25°. The endocardial and epicardial contours of the left ventricle were traced by semi-automatic contour detection in combination with manual interpretation and correction on all phases and slices using a standard software (EasyVision, release 4.4, Philips). The first frame was defined according to the starting point of the R wave in the ECG as end-diastole and the frame with the lowest blood volume was defined as end-systole. Absolute measurements were performed in a blinded manner with respect to the patient ID and the time of the examination.

On the basis of each cardiac MRI, the following individual heart parameters were calculated: LVM, end-diastolic volume (EDV), and end-systolic volume (ESV). HR and BP were measured during the MRI procedure. Calculations of CO, stroke volume (SV), and LVEF were performed based on the measures of EDV, ESV, and HR. The same, blinded observer evaluated all cardiac MRI data.

BP was measured twice after 10 min resting, and the mean values were used for calculating mean arterial pressure (MAP). MAP was calculated by the following formula: MAP=DBP+1/3×(SBP−DBP), where DBP is diastolic BP and SBP is systolic BP. Height and weight were recorded and the body surface area of each subject was calculated using the following formula: body surface area=0.007184×height^0.725^×weight^0.425^, where body surface area is measured in m^2^, height in cm, and weight in kg. The following parameters were corrected for body surface area: EDV, ESV, LVM, CO, and SV, whereas LVEF, HR, and MAP were not corrected. The corrected parameters are expressed as an index.

Thyroid hormones were measured by routine methods: total T_4_ and T_3_ concentrations, T_3_-uptake test, and TSH. Free T_4_ and T_3_ indices (FT_4_I and FT_3_I) were calculated as total T_4_ (T_3_) multiplied by the T_3_-uptake test. Reference ranges for the different assays were: TSH, 0.2–4.0 mU/l; FT_3_I, 0.8–2.4 (arbitrary units); and FT_4_I, 50–150 (arbitrary units).

This study was approved by the Local Ethical Committee, Region Copenhagen, Denmark (ID: H-4-2010-001), and all subjects gave informed consent after receiving written and oral information about the nature of the project.

### Statistical analysis

Paired and unpaired *t*-tests were used for evaluation. All calculations were performed using the statistical program SPSS version 20 (IBM Corp., Armonk, NY, USA).

## Results

Thyroid function parameters, BMI, and BP results for SH, before and after radioactive iodine as well as in the control group are given in Table 1. Besides TSH values, all parameters in Table 1 are normally distributed. Untreated subclinical hyperthyroid subjects had higher FT_3_I than controls. Radioactive iodine treatment with normalization of serum TSH levels resulted in a decrease in FT_4_I and FT_3_I, and an increase in BMI and body surface area.

All subjects obtained euthyroidism after one radioactive iodine dose, and the average radioactive iodine dose given to restore euthyroidism was (median and range) 407 (390–594) MBq. Time from radioactive iodine treatment to normalization of serum TSH levels was 87 (35–130) days. Time from normalization of serum TSH levels to second MRI evaluation was 139 (30–304) days.

Cardiac MRI data are given in Table 2, and these parameters are normally distributed. As the body surface area increased significantly after radioactive iodine treatment, we have included both the raw data as well as data corrected to body surface area when applicable. However, this did not change the overall findings. Comparing untreated subclinical hyperthyroid patients with controls, we found that SH had increased HR as well as CI. Treating SH with radioactive iodine resulted in a significant decrease in LVMI of mean 2.7 g/m^2^ (5%), in HR of 8 bpm (10%), and in CI of 0.24 l/min per m^2^ (7%), and an increase in end-diastolic volume index (EDVI) of 2.6 ml/m^2^ (5%). LVEF remained unchanged.

## Discussion

MRI is generally considered to be the most valid method to obtain functional and structural cardiac measures *in vivo* due to its high reproducibility and accuracy (9). Compared with echocardiography, cardiac MRI has two major advantages: first, cardiac MRI is observer independent during the acquisition of images of the heart, whereas, during echocardiography, appropriate ultrasonic images are obtained and chosen by the observer. Secondly, the precision of cardiac MRI is considerably greater as evaluated by both intra- and inter-observer variations. Studies based on cardiac MRI will therefore benefit from higher statistical power, and sample sizes required to detect significant differences are much smaller than studies based on echocardiography. Thus, it has been calculated that, to detect a clinical relevant change in LVM in a randomized, placebo-controlled study, less than one-twentieth the number of subjects is necessary using cardiac MRI when compared with echocardiography (16).

The main findings of this study were: i) patients with SH seem to have increased resting HR and CO, in this study as much as ∼25% and ii) radioactive iodine treatment to SH obtaining normal TSH levels and thus euthyroidism resulted in significant reductions in LVMI, HR, and CI in subclinical hyperthyroid patients after radioactive iodine treatment, and resting HR normalized when compared with controls. The reductions observed in LVMI and HR can be regarded as beneficial and clinically relevant. Several studies have demonstrated that regression of left ventricular hypertrophy is related to lower risk of cardiovascular events. Thus, initiating antihypertensive treatment with an angiotensin-converting-enzyme (ACE) inhibitor resulted in a reduction in ECG-evaluated left ventricular hypertrophy, which was associated with a concomitant reduction in both cardiovascular death and events (17), and ECG-evaluated left ventricular hypertrophy reductions in patients with resistant hypertension have been associated with a lower risk of cardiovascular events (18). With regard to the increased HR observed in SH, both epidemiological and observational studies suggest a link between elevated HR and increased risk of cardiovascular events among healthy individuals (19, 20). Moreover, treatment with the negative chronotropic drug Ivabradine seems to reduce the incidence of cardiovascular events in patients with left ventricular dysfunction due to coronary heart disease and a resting HR above 70 b.p.m. (21).

We have previously found significant decreases in HR and CO of a similar magnitude in radioactive iodine-treated subclinical hyperthyroid patients using another imaging technology, impedance cardiography (7). This particular method is also observer independent and has a relatively low CV, however, not to a magnitude as observed using MRI. Similar findings were obtained in a recent study by Kaminski *et al*. (15) in 44 patients with SH studied before and after radioactive iodine treatment using echocardiography technology. They found significant decreases in several parameters including a 9% reduction in LVMI, and 18% reduction in CI. On one parameter, their findings differed from ours, as they found a decrease in left ventricle EDVI in contrast to our finding of a small increase. Our result of an increase in EDVI might be explained by the rather pronounced decrease in HR. This might very well be the primary effect of a decrease in thyroid hormone availability in heart tissue as a consequence of obtaining euthyroidism, as it is well known that thyroid hormones have a pronounced chronotropic effect on the sinus node.

In this study, HR and therefore CI did not completely return to levels observed in the control group. This is not easy to interpret as both FT_4_I and FT_3_I decreased to normal values, and even to values lower than the means among controls. The reason for this finding might be due to the relatively small number of subjects in the groups under study.

The post-treatment cardiac MRI was performed in median 139 days after the first recorded normalization of TSH levels. This figure is probably somewhat underestimated as we did not measure TSH levels with especially short intervals, typically only with 2–3 months interval. Thus, the time period where the patients could be regarded as euthyroid with a reestablished pituitary–thyroid axis was probably longer than the reported 139 days. This might be of importance as one might argue that the time for normalization of remodeling of the heart after removing the increased burden on the heart (i.e. SH) resulting in a decrease in LWMI is relatively short in this study.

Whether the demonstrated reductions in HR and CO and thus heart load, which probably are the reasons for the small but significant reduction in LVMI, can be translated into a reduced burden of cardiovascular disease in the future has not been demonstrated in prospective trials. However, it is well known that SH is associated with increased risk of atrial fibrillation, MACEs, and increased all cause mortality (14). Therefore, we recommend on the basis of this and other studies that SH should be regarded as a disease that needs to be treated, and not just a period of transition into overt hyperthyroidism, during which an attitude of observation without treatment can be used.

### Limitations

We included a control group, but we did not repeat cardiac MRI in this group during follow-up. This could otherwise serve to reduce spontaneous changes in the measured cardiac parameters over time. In line with this, a randomized study would have been preferable. However, as the indication for radioactive iodine treatment was based on a combination of the finding of SH and compression symptoms from their goiter, which is a common practice in our institution, we denied from performing a placebo-controlled randomized study.

The investigated subjects in our study were in mean 59 years old, predominantly women, and did not suffer from other chronic diseases. This means that extrapolation of our results to people of younger age, male sex, or people with co-morbidity should be done with caution.

Strengths of this study were as follows: the inclusion of a homogenous group of subclinical hyperthyroid patients with a nodular goiter in a prospectively designed, comparison of the same patients before and after treatment; we used cardiac MRI technology, which can be considered as the gold standard method for evaluating cardiac function and structure.

## Conclusion

Our results based on cardiac MRI point to the fact that women in the age range of 44–71 years with untreated SH and a nodular goiter and no co-morbidity have higher resting HR and CO than euthyroid subjects. When these patients were rendered euthyroid by radioactive iodine treatment, clinically meaningful reductions in the load of the heart were achieved with reductions in HR and CO and a small but significant reduction in LVMI. These findings support that, in these patients, SH is a condition in which aggressive treatment should be considered to protect cardiac function.

## Author contribution statement

P D Mark performed the statistical analysis and drafted the manuscript. M Andreassen was responsible for evaluation of the control subjects. C L Petersen was responsible for running and analyzing the cardiac MRI. A Kjaer helped to run and analyze the cardiac MRI. J Faber has devised the study, participated in the design and coordination, and helped to draft the manuscript.

## Figures and Tables

**Table 1:** Thyroid function, body proportion, and blood pressure. Thyroid function parameters, BMI, body surface area (BSA), and mean arterial blood pressure (MAP) in subclinical hyperthyroidism (SH), before/after radioactive iodine (RAI) treatment, as well as in controls at baseline (mean±s.d. except TSH values given in median (range)).

**Parameter**	**Matched controls** (*n*=8)	**SH group** (*n*=12)		***P* value SH before/after RAI**
Before treatment	After treatment
BMI (kg/m^2^)	25.0±4.5	26.7±5.5	27.3±6.0	**0.021**
BSA (m^2^)	1.80±0.19	1.85±0.13	1.86±0.14	**0.029**
TSH (mU/l)	2.07 (0.80–3.20)	0.01 (0.01–0.09)	0.88 (0.27–3.99)	
FT_3_I (arb. units)	1.64±0.18	2.01±0.40*	1.30±0.20^†^	**<** **0.001**
FT_4_I (arb. units)	94.7±21.3	106±28.7	77.5±11.0*	**0.005**
MAP (mmHg)	95.3±13.1	101.3±5.6	101.1±5.5	0.920

Comparison to controls: *P<0.05; ^†^ *P*<0.01; arb., arbitrary.

**Table 2:** Cardiac MRI parameters. MRI data on subclinical hyperthyroidism (SH) subjects before/after radioactive iodine (RAI) treatment as well as in controls at baseline (mean±s.d.).

**Parameter**	**Matched controls** (*n*=8)	**SH group** (*n*=12)		***P* value SH before/after RAI**
Before treatment	After treatment
LVMI (g/m^2^)	54.1±6.7	54.4±8.7	51.8±9.2	**0.034**
LVM (g)	97.8±20.6	101±18.2	96.5±17.8	**0.048**
EDVI (ml/m^2^)	63.3±9.9	63.4±7.4	66.0±7.8	**0.013**
EDV (ml)	113±13.3	118±19.5	124±21.2	**0.005**
ESVI (ml/m^2^)	20.2±5.4	20.5±3.4	21.6±3.2	0.249
ESV (ml)	35.8±7.8	38.2±7.5	40.5±8.0	0.198
HR (b.p.m.)	65±16	81±10^†^	73±10	**0.001**
SVI (ml/m^2^)	43.1±7.3	42.9±6.1	44.4±7.0	0.092
SV (ml)	76.9±11.4	79.8±14.9	83.2±16.7	0.066
CI (l/min per m^2^)	2.72±0.42	3.48±0.55^†^	3.24±0.56*	**0.017**
CO (l/min)	4.86±0.67	6.46±1.19^†^	6.04±1.16*	**0.018**
LVEF (%)	68.4±6.6	67.5±4.3	66.5±3.7	0.417

Comparison with controls: *P<0.05; ^†^ *P*<0.01; EDSI, end-diastolic volume index; ESVI, end-systolic volume index.
